# Relationship Between Tooth Loss Due to Chronic Periodontitis and Lower Urinary Tract Symptoms

**DOI:** 10.3390/medicina60111829

**Published:** 2024-11-07

**Authors:** Tomohiro Matsuo, Shota Kakita, Hiroyuki Honda, Shintaro Mori, Kyohei Araki, Kensuke Mitsunari, Kojiro Ohba, Yasushi Mochizuki, Ryoichi Imamura

**Affiliations:** Department of Urology, Graduate School of Biomedical Sciences, Nagasaki University, Nagasaki 852-8501, Japan; s-kakita@nagasaki-u.ac.jp (S.K.); h.honda2306@nagasaki-u.ac.jp (H.H.); s-mori@nagasaki-u.ac.jp (S.M.); araki.k@nagasaki-u.ac.jp (K.A.); kmitsunari@nagasaki-u.ac.jp (K.M.); ohba-k@nagasaki-u.ac.jp (K.O.); mochi@nagasaki-u.ac.jp (Y.M.); ryo-imamura@nagasaki-u.ac.jp (R.I.)

**Keywords:** lower urinary tract symptoms, overactive bladder, tooth loss, chronic periodontitis

## Abstract

*Background and Objectives*: This study aimed to investigate the relationship between lower urinary tract symptoms (LUTS) and tooth loss due to chronic periodontitis. *Materials and Methods*: A total of 232 patients aged 40 years or older with LUTS were evaluated. The number of remaining teeth and the severity of LUTS were assessed using the overactive bladder symptom score, international prostate symptom score, and urodynamic tests. Statistical analyses, including multivariate and propensity score matching, were conducted to determine the relationship between tooth loss and overactive bladder (OAB). *Results*: Compared with the non-OAB group, the OAB group had significantly fewer remaining teeth (*p* < 0.001). A negative correlation was observed between the number of remaining teeth and the severity of LUTS, with symptoms such as urgency, frequency, and nocturia being more severe in patients with fewer teeth (all *p* < 0.001). Objective measures, including bladder capacity and maximum flow rate, were also significantly lower in patients with fewer teeth. Additionally, having fewer than 21 teeth was identified as a significant risk factor for LUTS. Multivariate analysis confirmed that tooth loss was an independent risk factor for OAB, even after adjusting for age and comorbidities (*p* < 0.001). Propensity score matching further validated the association between the number of remaining teeth and OAB (*p* = 0.022), highlighting a clear connection between dental health and urinary function. *Conclusions*: Tooth loss due to chronic periodontitis is strongly associated with the severity of LUTS, including storage and voiding symptoms. Maintaining good oral health may help reduce the risk of OAB and related urinary symptoms. This study underscores the importance of dental care in managing and preventing LUTS, suggesting that improving oral health could play a key role in mitigating these conditions.

## 1. Introduction

Overactive bladder (OAB) is a symptom-based syndrome characterized by urinary urgency, with or without urge incontinence [1]. Its prevalence is reported to be between 16% and 19% [2,3]. A recent large-scale epidemiological study in Japan revealed that the incidence of OAB increases with age [4]. The occurrence of OAB is closely associated with metabolic syndrome (MetS) and lifestyle-related diseases. The severity of OAB is believed to worsen with the degree of chronic ischemia caused by MetS and lifestyle diseases and the extent of chronic inflammation, both systemic and local, in the bladder [5,6]. Additionally, it is well established that lower urinary tract symptoms (LUTS), including OAB, significantly impact patients’ quality of life (QoL) [7].

In contrast, chronic periodontitis is a leading cause of tooth loss in adults, particularly in older adults [8]. In the field of dental health, the occurrence and severity of chronic periodontitis have been linked to MetS and lifestyle-related diseases [9,10]. Recent studies have shown that tooth loss due to chronic periodontitis is strongly associated with the development of various systemic diseases [11,12].

However, few studies have examined the relationship between LUTS, including OAB, and tooth loss. This study aimed to evaluate the correlation between OAB and periodontal health, specifically focusing on the number of remaining teeth. Additionally, this study sought to investigate whether the number of remaining teeth is a potential risk factor for the onset of OAB and to clarify the relationship between the severity of OAB and the number of remaining teeth.

## 2. Materials and Methods

### 2.1. Patients and Study Design

**Figure 1:**
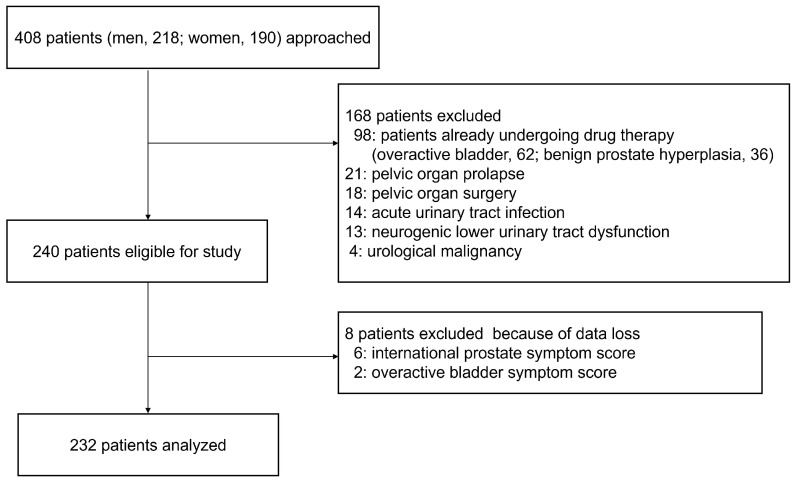
Patient flow diagram.

Our study enrolled new patients (aged ≥40 years) who presented to Nagasaki University Hospital with at least one LUTS between January 2021 and March 2023. A total of 408 (218 males and 190 females) consecutive patients who met the inclusion criteria were included. Patients already receiving treatment for OAB, benign prostatic hyperplasia, acute urinary tract infections, or any conditions affecting urinary function, such as a history of pelvic surgery, pelvic organ prolapse, urological malignancy, or neurogenic lower urinary tract dysfunction, were excluded (Figure 1). We recorded the number of remaining teeth and examined its association with LUTS. The patients were categorized into two groups based on the presence (OAB group) or absence (non-OAB group) of OAB. We then investigated the association between the number of teeth remaining due to chronic periodontitis, diagnosed by primary care dentists, and LUTS, including OAB. Additionally, participants were categorized into three groups based on their OAB symptom score (OABSS) as follows: non-OAB (0–2), mild OAB (3–5), and moderate/severe OAB (6–15). Differences in LUTS and the number of remaining teeth were analyzed across the three groups.

This study was approved by the Nagasaki University Hospital Ethics Committee (#20122138) and conducted in accordance with the principles of the Declaration of Helsinki. Written informed consent was obtained from all participants. Based on the OABSS, participants with urinary urgency (Question [Q] 3 score ≥2 and total score ≥3) were considered to have OAB [13].

### 2.2. Evaluation of the Number of Remaining Teeth, Subjective Symptoms, and Objective Findings in Patients

Two urologists (S.M. and H.H.) counted the number of remaining teeth, excluding dental implants and dentures. Subjective LUTS were assessed using the OABSS and international prostate symptom score (IPSS). Objective findings were evaluated using uroflowmetry and post-void residual urine (PVR). The voided volume (VV) and maximum flow rate were measured using free uroflowmetry with the AQUARIUS^®^ CTS (Laborie Medical Technologies, Corp., Portsmouth, NH, USA). PVR was assessed using transabdominal ultrasound sonography (HI VISION Avius^®^, Hitachi-Aloka Medical, Ltd., Tokyo, Japan).

### 2.3. Statistical Analyses

All statistical data are presented as the mean ± standard deviation (SD). Differences in subjective and objective symptoms and the number of remaining teeth between the OAB and non-OAB groups were evaluated using the Student’s *t*-test and Mann–Whitney U test, as appropriate. The Kruskal–Wallis test was applied to analyze the relationship between OAB severity and the number of remaining teeth, with multiple comparisons conducted using the Steel–Dwass method. Spearman’s rank correlation coefficient (ρ) was employed to assess correlations between continuous variables, and corresponding *p*-values were reported. Logistic regression analysis was used to estimate crude and adjusted effects, expressed as odds ratios (ORs) with 95% confidence intervals (CIs) and *p*-values.

To control for potential confounding factors in the relationship between tooth loss and the risk of overactive bladder (OAB), we adjusted for specific variables in our logistic regression model. Specifically, we included age, as it is a well-established risk factor for both OAB and periodontal health. Additionally, adjustments were made for systemic comorbidities, such as chronic kidney disease, hypertension, and diabetes mellitus, as these conditions may independently influence the severity of OAB symptoms. By including these variables, we aimed to ensure that the observed association between tooth loss and OAB was not confounded by other underlying health conditions.

All tests were two-sided, and a *p*-value of <0.05 was considered statistically significant. Statistical analyses were conducted using JMP 17 software (SAS Institute Inc., Cary, NC, USA). The study sample size was determined using G*Power version 3.1 software based on prior studies [14,15], with a two-sided probability of 0.05, a power of 80%, and an effect size of 0.5, estimating the ideal sample size to be 224 participants.

### 2.4. Propensity Score Matching

Patients with OAB were matched 1:1 with those without OAB based on their propensity scores using nearest-neighbor matching. A caliper width of 0.2 SDs was used.

## 3. Results

### 3.1. Patients’ Characteristics and Differences in Urological Parameters Between OAB and Non-OAB Groups

Table 1 presents the patient characteristics in this cross-sectional study. Although the ideal sample size calculated with G*Power was 224, as described above, we ultimately included 232 participants in the study to strengthen the reliability of the results. Of the 232 participants included in the analysis, 129 (55.6%) were male patients. The overall mean age was 67.6 ± 10.9 years. Of the 232 patients, 100 (43.1%) met the diagnostic criteria for OAB according to the OABSS. The mean age of patients was significantly higher in the OAB group than in the non-OAB group (*p* = 0.001). Additionally, all individual question scores and the total OABSS were higher in the OAB group than in the non-OAB group (all *p* < 0.001). Both IPSS storage and voiding symptoms were elevated in the OAB group than in the non-OAB group, along with a significantly lower QoL (all *p* < 0.001) (Table 1). However, no significant differences in comorbidities, including hypertension, diabetes mellitus, and hyperlipidemia, were observed between the two groups.

### 3.2. Differences in the Number of Remaining Teeth Between OAB and Non-OAB Groups

The number of patients with chronic periodontitis and those wearing dentures was significantly higher in the OAB group than in the non-OAB group (both *p* < 0.001). Additionally, the number of remaining teeth was significantly lower in the OAB group (12.8 ± 8.8) than in the non-OAB group (21.5 ± 8.5) (*p* < 0.001) (Table 1).

### 3.3. Relation Between OAB Severity and Systematic Comorbidities and the Number of Remaining Teeth

Table 2 presents a comparative analysis of the three groups classified by OAB severity. The mean age of patients in the moderate/severe group was highest among the three groups (*p* = 0.005). Although no significant difference in the prevalence of systemic comorbidities was observed between the groups, the prevalence of chronic periodontitis and denture use was significantly associated with OAB severity (both *p* < 0.001). The number of remaining teeth was lowest in the moderate/severe group (*p* < 0.001). Additionally, both OABSS and IPSS correlated with OAB severity. Regarding objective findings, although no significant differences were observed in PVR among the three groups, VV and Qmax were lowest in the moderate/severe group (both *p* < 0.001).

### 3.4. Correlation Between the Number of Remaining Teeth and OABSS

Table 3 presents the relationship between the number of remaining teeth and LUTS. In terms of subjective symptoms, a statistically significant negative correlation was observed between the number of remaining teeth and the individual question scores and total OABSS (all *p* < 0.001). Similarly, a significant negative correlation was observed between the number of remaining teeth and urinary symptoms in the IPSS, except for Q3 (intermittency), Q5 (weak stream), and Q6 (straining). Among the questions in the OABSS and IPSS, nighttime frequency (OABSS Q2, IPSS Q7) and urgency (OABSS Q3, IPSS Q4) showed the strongest association with the number of remaining teeth. Additionally, the number of teeth was significantly associated with patients’ QoL due to LUTS (IPSS-QoL, ρ = −0.464, *p* < 0.001). In terms of objective findings, VV (ρ = 0.303, *p* < 0.001) and Qmax (ρ = 0.219, *p* < 0.001) showed a positive correlation with the number of remaining teeth; however, no statistically significant correlation was observed with PVR (ρ = −0.125, *p* = 0.058).

### 3.5. Predictive Marker for the Presence of OAB

To explore the relationship between OAB and the number of remaining teeth, we conducted a receiver operating characteristic curve analysis. The optimal cutoff for the number of remaining teeth was determined to be 21 (area under the curve, 0.757; sensitivity, 78.0%; specificity, 78.4%; *p* < 0.001). Based on these results, we performed both univariate and multivariate analyses to identify risk factors for OAB. The number of remaining teeth emerged as the only significant risk factor for OAB in both analyses (Table 4). Additionally, we assessed whether the number of remaining teeth could serve as a predictor of OAB using propensity score matching (Table 5 and Table 6). This analysis included data from 130 patients (65 in each cohort). The standardized mean differences for all characteristics were less than 0.15, indicating minimal baseline differences between the groups (Table 5). Patients with fewer than 21 remaining teeth were significantly more likely to belong to the OAB group (*p* = 0.022; OR, 2.278; 95% CI, 1.122–4.630) (Table 6).

## 4. Discussion

In this study, we found that the OAB group had a significantly higher number of older patients, those with chronic periodontitis, and those with fewer remaining teeth compared with those in the non-OAB group. Additionally, the severity of OAB was associated with age and dental health, particularly the number of remaining teeth. The number of remaining teeth was negatively correlated with storage and voiding symptoms, which influenced bladder capacity and urinary flow, as observed through objective findings. Using detailed statistical methods such as multivariate analysis and propensity score matching, this study revealed a strong association between the number of remaining teeth and OAB prevalence.

A correlation between OAB prevalence and age was observed in this study. Objective findings indicated that bladder capacity and urinary flow were lower in the OAB group than in the non-OAB group; however, PVR showed no difference between them. These results, both in terms of subjective symptoms and objective findings, are consistent with previous studies [4,16]. Additionally, this study found correlations between OAB and dental health indicators, such as the prevalence of chronic periodontitis, rate of denture use, and number of remaining teeth. Compared with the non-OAB group, the OAB group exhibited a higher rate of denture use and fewer remaining teeth. Previous epidemiological studies have extensively documented the associations among age, chronic periodontitis, denture use, and tooth loss [17,18]. Moreover, our findings suggest that age itself may affect the onset of OAB and periodontal health in the OAB group compared with the non-OAB group.

In contrast, no association was found between the incidence or severity of OAB and the prevalence of common lifestyle-related diseases in the present study. Although BMI was higher in the moderate/severe group than in the mild group, this difference was not statistically significant. In general, MetS and lifestyle-related diseases are strongly associated with the development of OAB, and some studies have suggested that an increase in MetS components contributes to OAB onset and severity [5,19,20]. In our study, the lack of association between systemic comorbidities and OAB onset or severity remains unclear. However, one possible explanation is the relatively high average age of the patients (67.6 years), with a large population of older adults.

Older adults are more prone to MetS and lifestyle-related diseases [21]. In fact, even in the non-OAB group, 44.7% of the patients had hypertension, a common systemic comorbidity in older adults. Additionally, many patients had other systemic diseases. This study did not account for how the treatment of systemic comorbidities might have affected OAB severity, nor did we have detailed information regarding the severity of these conditions. Furthermore, because the sample size was not initially calculated to support sub-analysis by OAB severity, conclusive results on the relationship between lifestyle-related diseases and OAB could not be obtained. For instance, although no statistically significant difference in the number of patients with diabetes or chronic renal dysfunction was observed between the groups, the number of patients with severe OAB increased. Despite this, as OAB severity increased, the prevalence of chronic periodontitis and denture use increased, and the number of remaining teeth decreased.

A significant negative correlation was observed between the number of remaining teeth and all individual item scores and total OABSS, with particularly strong correlations between nocturia and urgency. However, when using the IPSS to assess voiding symptoms, a correlation between the number of remaining teeth and LUTS severity was only found for the sensation of incomplete emptying. A statistically significant, albeit weak, correlation was also observed between the number of remaining teeth and the voiding symptom score from the IPSS. Additionally, a correlation was observed between the number of remaining teeth and both VV and Qmax in the objective findings. This study further clarified that the number of remaining teeth was a risk factor for OAB, as confirmed through both univariate and multivariate analyses.

Several studies have investigated the relationship between chronic periodontitis and the incidence of LUTS. Matsumoto et al. distributed a self-assessment checklist for chronic periodontitis and a questionnaire on LUTS to 600 adult men and women who were first-time visitors to a dental clinic and collected their responses [22]. The authors found that chronic periodontitis was associated with urgency score on the OABSS, particularly in men, and that the severity of chronic periodontitis correlated with urinary urgency. However, no association was observed between chronic periodontitis and OAB symptoms in women. Conversely, voiding symptoms moderately correlated with chronic periodontitis in both men and women. The primary difference between their study and ours lies in the patient population, as their study included individuals visiting a dental clinic, whereas ours focused on patients from a urological clinic. Moreover, while the previous study elucidated the relationship between chronic periodontitis and LUTS, our study highlighted the association between the number of remaining teeth and LUTS as the primary outcome. In the previous study, no correlation was observed between the presence or severity of chronic periodontitis and OAB symptoms in women. By contrast, our study found that tooth loss due to chronic periodontitis was associated with both the presence and severity of OAB symptoms.

Additionally, a recent cross-sectional study including local residents reported a relationship between oral frailty, represented by chronic periodontitis, and LUTS [23]. Oral frailty is also associated with systemic vulnerability, and it is hypothesized that chronic periodontitis induces abnormally high oxidative stress, both locally and systemically, contributing to the onset and exacerbation of LUTS, including OAB [23]. In more detail, periodontitis, a chronic inflammatory condition that is a leading cause of tooth loss, can have systemic effects through mechanisms such as bacteremia, release of inflammatory mediators, and oxidative stress [24,25]. These processes contribute to systemic inflammation, potentially impacting various organ systems [26]. Specifically, periodontal pathogens trigger the production of pro-inflammatory cytokines, such as interleukin-1β, tumor necrosis factor-α, and interleukin-6 [26]. Oxidative stress in periodontitis involves the generation of reactive oxygen species, such as superoxide anion and hydrogen peroxide, overwhelming antioxidant defenses, such as superoxide dismutase and catalase [27]. While the link between periodontitis and conditions such as cardiovascular disease is well established [25,27], its influence on LUTS remains understudied. However, plausible mechanisms exist, including systemic inflammation, oxidative stress, and vascular effects, which could theoretically affect bladder function [26]. For instance, elevated levels of circulating inflammatory markers, such as C-reactive protein and matrix metalloproteinases, could potentially impact bladder tissue [26]. Further research is warranted to elucidate the potential connection between periodontal health and LUTS, emphasizing the importance of oral health in overall well-being and the need for interdisciplinary approaches in healthcare [25,27]. Despite these studies, the relationship between the oral environment and objective LUTS findings remains unclear. The present study is the first to employ urodynamic testing to elucidate the relationship between the number of remaining teeth and objective findings related to LUTS.

Recent prospective studies have demonstrated a strong relationship between the number of teeth and the incidence and prognosis of cardiovascular disease [28] and diabetes [29]. Moreover, chronic periodontitis and tooth loss appear to be associated with various malignant tumors [30,31]. Oral health-related conditions, including OAB, have also been implicated in the development of LUTS. However, it is important to highlight that our study revealed that the number of remaining teeth significantly impacts the occurrence and severity of LUTS, independent of the presence of systemic diseases.

The limitations of this study include its small sample size, relatively high proportion of older adult patients, cross-sectional design, focus on patients visiting the urology department, lack of information regarding the treatment of systemic comorbidities, and unclear severity of chronic periodontitis. Hence, the sample predominantly consists of older adults, which may limit the generalizability of the findings to other age groups. Furthermore, we were unable to collect detailed information on the treatment of systemic comorbidities, which may influence OAB symptoms. Although no significant association was found between lifestyle-related diseases, such as hypertension and diabetes, and the severity of OAB, it remains uncertain whether this lack of association was attributed to the older age of our sample or the primary focus on tooth count and OAB. Further studies are warranted to clarify these factors. However, this study reflects real-world clinical practice, as LUTS are more prevalent among older adult patients.

It is well established that the risk of tooth loss increases with the progression of chronic periodontitis, with more severe cases posing a higher risk [32]. The results of this study strongly suggest that the likelihood of developing LUTS as a symptom of systemic comorbidities may increase in patients with significant tooth loss. Therefore, large-scale and long-term prospective studies involving both urology and dental departments are necessary to determine whether oral health management can effectively prevent the onset of LUTS.

## 5. Conclusions

This study revealed that tooth loss due to chronic periodontitis may serve as a potential indicator of increased risk for LUTS, particularly in relation to the onset and severity of storage and voiding symptoms. Although a direct causative link cannot be conclusively established, monitoring the number of remaining teeth may help identify patients at higher risk for LUTS. Further research is warranted to explore whether interventions that improve oral health might reduce the incidence or alleviate the severity of LUTS.

## Figures and Tables

**Table 1:** Patients’ Background.

	Overall	Non-OAB	OAB	*p*-Value
Number of patients; *n* (%)	232 (100)	132 (56.9)	100 (43.1)	-
Gender (male, %)	129 (55.6)	73 (55.3)	56 (56.0)	0.916
Age (years)	67.6 ± 10.9	65.7 ± 10.9	70.1 ± 10.5	0.001
BMI (kg/m^2^)	22.3 ± 4.4	22.8 ± 3.8	23.9 ± 5.2	0.065
Comorbidity				
Hypertension (%)	112 (48.3)	59 (44.7)	53 (53.0)	0.234
Diabetes Mellitus (%)	38 (16.4)	18 (13.6)	20 (20.0)	0.213
Chronic kidney disease (%)	80 (34.5)	41 (31.1)	39 (39.0)	0.213
Hyperlipidemia (%)	58 (25.0)	33 (25.0)	25 (25.0)	1.000
Chronic periodontitis (%)	168 (72.4)	80 (60.6)	88 (88.0)	<0.001
Wearing dentures (%)	94 (40.5)	32 (24.4)	62 (62.0)	<0.001
Number of the remaining teeth	17.8 ± 9.6	21.5 ± 8.5	12.8 ± 8.8	<0.001
Urological parameters				
OABSS				
Q1 daytime frequency	0.6 ± 0.7	0.3 ± 0.5	1.0 ± 0.7	<0.001
Q2 nighttime frequency	1.8 ± 1.1	1.3 ± 1.1	2.3 ± 0.9	<0.001
Q3 urgency	1.4 ± 1.3	0.4 ± 0.5	2.6 ± 0.9	<0.001
Q4 urgency (incontinence)	0.4 ± 0.9	0.1 ± 0.2	0.8 ± 1.3	<0.001
Total OABSS	4.1 ± 3.0	2.1 ± 1.6	6.7 ± 2.4	<0.001
IPSS				
Q1 incomplete emptying	1.2 ± 1.3	0.9 ± 1.2	1.6 ± 1.4	<0.001
Q2 daytime frequency	1.0 ± 1.1	0.5 ± 0.8	1.7 ± 1.2	<0.001
Q3 intermittency	0.8 ± 1.2	0.6 ± 1.0	1.1 ± 1.3	0.002
Q4 urgency	1.5 ± 1.4	0.7 ± 0.9	2.6 ± 1.2	<0.001
Q5 weak stream	1.1 ± 1.4	0.8 ± 1.2	1.4 ± 1.5	<0.001
Q6 straining	0.6 ± 1.0	0.4 ± 0.8	0.9 ± 1.1	<0.001
Q7 nocturia	2.1 ± 1.4	1.4 ± 1.2	2.9 ± 1.2	<0.001
Storage symptoms (Q2 + Q4 + Q7)	4.6 ± 3.2	2.7 ± 2.1	7.2 ± 2.5	<0.001
Voiding symptoms (Q1 + Q3 + Q5 + Q6)	3.7 ± 4.0	2.7 ± 3.4	5.0 ± 4.4	<0.001
Total IPSS	8.3 ± 5.9	5.3 ± 4.6	12.2 ± 5.2	<0.001
IPSS-QoL score	3.1 ± 1.6	2.2 ± 1.3	4.2 ± 1.2	<0.001
Objective symptoms				
VV (mL)	242.0 ± 107.8	275.9 ± 101.7	197.1 ± 99.1	<0.001
Qmax (mL/s)	18.4 ± 9.0	20.5 ± 8.1	16.1 ± 9.7	<0.001
PVR (mL)	30.7 ± 28.1	30.3 ± 26.7	31.3 ± 30.1	0.789

OAB, overactive bladder; BMI, body mass index; OABSS, overactive bladder symptom score; IPSS, international prostate symptom score; QoL, quality of life; VV, voided volume; Qmax, maximum flow volume; PVR, post-void residual urine.

**Table 2:** Relationship between systematic comorbidities and the number of remaining teeth.

	Non-OAB	Mild OAB	Moderate/Severe OAB	*p*-Value
Number of patients; *n* (%)	132 (56.9)	35 (15.1)	65 (28.0)	-
Sex (male, %)	73 (55.3)	21 (60.0)	35 (53.8)	0.835
Age (years)	65.7 ± 10.9	69.0 ± 12.0	70.8 ± 9.6 **	0.005
BMI (kg/m^2^)	22.8 ± 3.8	23.2 ± 5.1	24.3 ± 5.2	0.075
Comorbidity				
Hypertension (%)	59 (44.7)	22 (62.9)	31 (47.7)	0.160
Diabetes Mellitus (%)	18 (13.6)	5 (14.3)	15 (23.1)	0.227
Chronic kidney disease (%)	41 (31.1)	11 (31.4)	28 (43.1)	0.228
Hyperlipidemia (%)	33 (25.0)	10 (28.6)	15 (23.1)	0.833
Chronic periodontitis (%)	80 (60.6)	29 (82.9) *	59 (90.8) **	<0.001
Wearing dentures (%)	32 (24.4)	20 (57.1) **	42 (64.6) **	<0.001
Number of the remaining teeth	21.5 ± 8.5	14.9 ± 9.2 **	11.7 ± 8.4 **	<0.001
Urological parameters				
OABSS				
Q1 daytime frequency	0.3 ± 0.5	0.6 ± 0.7 **	1.2 ± 0.5 **^,††^	<0.001
Q2 nighttime frequency	1.3 ± 1.1	1.6 ± 0.8	2.7 ± 0.6 **^,††^	<0.001
Q3 urgency	0.4 ± 0.5	2.2 ± 0.4 **	2.8 ± 1.0 **^,††^	<0.001
Q4 urgency (incontinence)	0.1 ± 0.2	0.1 ± 0.3	1.1 ± 1.4 **^,††^	<0.001
Total OABSS	2.1 ± 1.6	4.5 ± 0.6 **	7.8 ± 2.2 **^,††^	<0.001
IPSS				
Q1 incomplete emptying	0.9 ± 1.2	1.3 ± 1.0 *	1.7 ± 1.5 **	<0.001
Q2 daytime frequency	0.5 ± 0.8	1.1 ± 0.8 **	2.1 ± 1.2 **^,††^	<0.001
Q3 intermittency	0.6 ± 1.0	0.8 ± 1.1	1.2 ± 1.4 **	0.004
Q4 urgency	0.7 ± 0.9	2.1 ± 1.0 **	2.9 ± 1.2 **^,††^	<0.001
Q5 weak stream	0.8 ± 1.2	1.3 ± 1.4	1.5 ± 1.6 **^,††^	0.002
Q6 straining	0.4 ± 0.8	0.7 ± 0.8	1.0 ± 1.3 **	0.001
Q7 nocturia	1.4 ± 1.2	2.0 ± 1.1 **	3.4 ± 1.0 **^,††^	<0.001
Storage symptoms (Q2 + Q4 + Q7)	2.7 ± 2.1	5.1 ± 1.5 **	8.3 ± 2.1 **^,††^	<0.001
Voiding symptoms (Q1 + Q3 + Q5 + Q6)	2.7 ± 3.4	4.2 ± 3.3 *	5.4 ± 4.9 **	<0.001
Total IPSS	5.3 ± 4.6	9.3 ± 3.4 **	13.7 ± 5.5 **^,††^	<0.001
IPSS-QoL score	2.2 ± 1.3	3.6 ± 1.2 **^,††^	4.6 ± 1.0 **^,††^	<0.001
Objective symptoms				
VV (mL)	275.9 ± 101.7	225.1 ± 96.4 **	182.1 ± 98.0 **^,†^	<0.001
Qmax (mL/s)	20.5 ± 8.1	17.4 ± 8.8	15.3 ± 10.1 **	<0.001
PVR (mL)	30.3 ± 26.7	37.3 ± 31.8	28.1 ± 29.0	0.168

OAB, overactive bladder; BMI, body mass index; OABSS, overactive bladder symptom score; IPSS, international prostate symptom score; QoL, quality of life; VV, voided volume; Qmax, maximum flow volume; PVR, post-void residual urine; * *p* < 0.05 vs. Non-OAB; ** *p* < 0.01 vs. Non-OAB; ^†^ *p* < 0.05 vs. Mild OAB; ^††^ *p* < 0.01 vs. Mild OAB.

**Table 3:** Relationship between lower urinary tract symptoms and the number of remaining teeth.

	ρ	*p*-Value
OABSS		
Q1 daytime frequency	−0.416	<0.001
Q2 nighttime frequency	−0.525	<0.001
Q3 urgency	−0.474	<0.001
Q4 urgency incontinence	−0.290	<0.001
Total OABSS	−0.572	<0.001
IPSS		
Q1 incomplete emptying	−0.205	0.002
Q2 daytime frequency	−0.335	<0.001
Q3 intermittency	−0.106	0.106
Q4 urgency	−0.426	<0.001
Q5 weak stream	−0.118	0.074
Q6 straining	−0.119	0.070
Q7 nocturia	−0.535	<0.001
Storage symptoms (Q2 + Q4 + Q7)	−0.540	<0.001
Voiding symptoms (Q1 + Q3 + Q5 + Q6)	−0.168	0.010
Total IPSS	−0.407	<0.001
IPSS-QoL score	−0.464	<0.001
Objective findings		
VV (mL)	0.303	<0.001
Qmax (mL/s)	0.219	<0.001
PVR (mL)	−0.125	0.058

OABSS, overactive bladder symptom score; IPSS, international prostate symptom score; QoL, quality of life; VV, voided volume; Qmax, maximum flow volume; PVR, post-void residual urine.

**Table 4:** Univariate and multivariate analyses for the predictive factors of OAB.

	Univariate Analysis			Multivariate Analysis		
	OR	95% CI	*p*-Value	OR	95% CI	*p*-Value
Sex (male)	1.029	0.610–1.739	0.916	-	-	-
Age	1.041	1.015–1.069	0.002	1.000	0.971–1.032	0.951
BMI	0.943	0.887–1.001	0.054	-	-	-
Hypertension	1.395	0.829–2.356	0.210	-	-	-
Diabetes Mellitus	1.583	0.782–3.206	0.197	-	-	-
Chronic kidney disease	1.420	0.822–2.452	0.208	-	-	-
Hyperlipidemia	1.000	0.545–1.818	1.000	-	-	-
Chronic periodontitis	4.767	2.444–9.949	<0.001	1.497	0.571–3.921	0.412
Wearing dentures	5.097	2.918–9.089	<0.001	1.743	0.791–3.921	0.158
Remaining teeth (<21)	7.028	3.953–12.865	<0.001	4.728	2.162–10.652	<0.001

OAB, overactive bladder; OR, odds ratio; CI, confidence interval, BMI, body mass index.

**Table 5:** Baseline characteristic data of OAB after propensity score matching.

	Non-OAB *n* = 65	OAB *n* = 65	*p*-Value	Standardized Mean Differences
Sex (male)	36 (55.4)	40 (61.5)	0.594	0.125
Age	68.2 ± 11.7	70.6 ± 10.4	0.217	0.117
BMI	23.9 ± 3.8	23.1 ± 4.5	0.276	0.112
Hypertension	31 (47.7)	37 (56.9)	0.380	0.126
Diabetes Mellitus	13 (20.0)	12 (18.5)	1.000	0.039
Chronic kidney disease	24 (36.9)	25 (38.5)	1.000	0.032
Hyperlipidemia	19 (29.2)	15 (23.1)	0.550	0.140
Chronic periodontitis	42 (64.6)	45 (69.2)	0.709	0.052
Wearing dentures	32 (49.2)	32 (49.2)	1.000	0.001

OAB, overactive bladder; BMI, body mass index.

**Table 6:** Differences in the number of remaining teeth between the OAB and non-OAB groups.

	Non-OAB *n* = 65	OAB *n* = 65	*p*-Value
Remaining teeth (<21)	30 (46.2)	43 (66.2)	0.022

OAB, overactive bladder.

## Data Availability

The data supporting this study are available from the corresponding author upon reasonable request. The data are not publicly accessible due to privacy and ethical considerations.
